# Culture Clash? Investigating constructions of sexual and reproductive health from the perspective of 1.5 generation migrants in Australia using Q methodology

**DOI:** 10.1186/s12978-017-0310-9

**Published:** 2017-04-04

**Authors:** T. Dune, J. Perz, Z. Mengesha, D. Ayika

**Affiliations:** 1 School of Science and Health, Western Sydney University, Penrith, Australia; 2 Centre for Health Research, School of Medicine, Western Sydney University, Locked Bag 1797, Penrith, NSW 2571 Australia

**Keywords:** Q methodology, 1.5 generation migrants, Sexual and reproductive health, Australia, Cross cultural

## Abstract

**Background:**

In Australia, those who migrate as children or adolescents (1.5 generation migrants) may have entered a new cultural environment at a crucial time in their psychosexual development. These migrants may have to contend with constructions of sexual and reproductive health from at least two cultures which may be at conflict on the matter. This study was designed to investigate the role of culture in constructions of sexual and reproductive health and health care seeking behaviour from the perspective of 1.5 generation migrants.

**Methods:**

Forty-two adults from various ethno-cultural backgrounds took part in this Q methodological study. Online, participants rank-ordered forty-two statements about constructions of sexual and reproductive health and health seeking behaviours based on the level to which they agreed or disagreed with them. Participants then answered a series of questions about the extent to which their ethnic/cultural affiliations influenced their identity. A by-person factor analysis was then conducted, with factors extracted using the centroid technique and a varimax rotation.

**Results:**

A seven-factor solution provided the best conceptual fit for constructions of sexual and reproductive health and help-seeking. Factor A compared progressive and traditional sexual and reproductive health values. Factor B highlighted migrants’ experiences through two cultural lenses. Factor C explored migrant understandings of sexual and reproductive health in the context of culture. Factor D explained the role of culture in migrants’ intimate relationships, beliefs about migrant sexual and reproductive health and engagement of health care services. Factor E described the impact of culture on sexual and reproductive health related behaviour. Factor F presented the messages migrant youth are given about sexual and reproductive health. Lastly, Factor G compared constructions of sexual and reproductive health across cultures.

**Conclusions:**

This study has demonstrated that when the cultural norms of migrants’ country of origin are maintained it has a significant influence on how 1.5 generation migrants construct, experience and understand various aspects of sexual and reproductive health. Policy makers, health care professionals and resettlement service providers are advised to engage with migrant parents and youth in exploring, discussing, reframing and reconstructing SRH in an Australian context.

## Plain English summary

In Australia, those who migrate as children or adolescents (1.5 generation migrants) may have entered a new cultural environment at a crucial time in their psychosexual development. These migrants may have to contend with constructions of sexual and reproductive health from at least two cultures which may be in conflict. This study, using Q methodology, investigated the role of culture in constructions of sexual and reproductive health from the perspective of 1.5 generation migrants. Analysis resulted in seven distinct yet interrelated factors. The findings of this study demonstrate that young migrants vary in their acculturation journeys. Notably, when the cultural norms of migrants’ country of origin persist post-migration it has a significant influence on how 1.5 generation migrants construct, experience and understand sexual and reproductive health. The findings indicate that as norms take on more cross-cultural constructions they can reshape to produce multicultural ways of understanding and experiencing sexual and reproductive health without young migrants having to lose their relationship with their culture of origin. Policy makers, health care professionals and resettlement service providers are advised to engage with migrant communities, parents and youth in exploring, discussing, reframing and reconstructing SRH in an Australian context.

## Background

Migration within an international context has increased exponentially especially in the past two decades [1]. Of particular note is the Australian context where over 27% of Australians were born overseas and another 20% have at least one parent born overseas [2]. Notably, net overseas migration contributes to over 60% of Australia’s total population growth [3]. Australia has also committed to the resettlement of over 12,000 new refugees in addition to the current 13,500 new refugees arriving annually [4]. Australia thus provides a particularly rich case study of a migrant-receiving country undergoing rapid transformation. Australia has also been found to have pockets of cultural concentration which allows migrants to stay connected to key aspects of their culture such as their ethnicity, community, language and religion [5, 6]. To that effect, it is likely that the maintenance and preservation of cultural and religious norms of a migrant’s country of origin have a significant influence on how migrants in this region construct, experience, understand and manage their health. This study was therefore designed to investigate the role of culture in constructions of sexual and reproductive health (SRH) and help-seeking behaviour from the perspective of 1.5 generation migrants.

### The influence of culture on sexual and reproductive health and help-seeking

Cultural differences between a migrant’s country of origin and that of immigration are linked to reduced help-seeking behaviour across a range of health outcomes [7] and especially with regard to sexual and reproductive health (SRH) [8, 9]. SRH is of particular note as many cultures have quite clear ideologies about sexuality, sexual behaviour and thus SRH [10]. Research indicates that when migrants feel bound to constructions of SRH as per their ethnic origins they may not utilise SRH services [8, 9]. Migrants may perceive these services to be inappropriate or that seeking such services would be perceived of negatively by their cultural group [10]. For migrants arriving from countries with very different cultural, ethnic and religious values and beliefs to those in Australia the process of adapting constructions, understandings and experiences of sexuality often results in a number of challenges [11].

Such differences and outcomes often relate to a wide range of SRH issues including, but not limited to, abortion, contraceptives, gender and gender equality, sexual intercourse and behaviour, sexual pleasure and satisfaction [12]. While these issues are integral to SRH and help-seeking it is the processes of construction which encompass these concepts which are of interest here. While much is known about the potential impact of culture on many aspects of SRH (e.g., contraceptive use, maternal health care or condom-use) little is known about the processes of construction that underlie migrants’ SRH experiences and decision making which develop during childhood and adolescence.

### 1.5 generation migrants and constructions of sexual and reproductive health and help-seeking

Here SRH refers to “a state of complete physical, mental and social well-being in all matters relating to the reproductive system. It implies that people are able to have a satisfying and safe sex life, the capability to reproduce, and the freedom to decide if, when, and how often to do so” [13]. Good SRH also includes SRH help-seeking and involves access and utilisation of accurate sexual health information; safe, effective, affordable and acceptable contraception methods; and maternal health support [13]. The ways in which people from various, let alone, cross/multicultural backgrounds construct and subsequently engage with SRH concepts is not universal.

This is of particular relevance to 1.5 generation migrants who are culturally from two worlds. 1.5 generation migrants are not conventional first generation migrants, who are often adults eligible to emigrate on their own, nor are they the conventional second generation migrant, the offspring of the first-generation migrant born in the adoptive country. 1.5 generation migrants are often expected to uphold (by other members of their cultural community) particular norms about SRH [14] while at the same time adopting and enacting Australian constructions of SRH [11]. Experiencing a culture clash in this context may have immediate and far-reaching implications for the SRH and help-seeking of 1.5 generation migrants.

Discourse around the experiences of 1.5 generation migrants, given their unique cross-cultural position, is increasing. Extant research has explored 1.5 generation migrants experiences of sociocultural identity [15, 16], effects of acculturation and discrimination on mental health [17], family reunification amongst Filipinos in France [18], belonging among diasporic African communities in the UK [19] and the hybridity and interculturality of 1.5 generation Chinese migrants in New Zealand [20]. Although research related to this cohort of migrants is emerging, few studies consider the nexus between 1.5 generation status and SRH. This is however an important area for consideration as cultural meanings and input are imbued into all elements of sexuality providing a significant framework for constructions of SRH and vice versa [21]. As Agocha, Asencio and Decena [22] explain, “the values, beliefs, and behaviours associated with sexuality reveals a great deal about the larger beliefs and values of the society they inhabit or from which they originate”. Considering 1.5 generation migrants’ cross-cultural positionality taking time to consider and manage the expectations of two cultures may result in delayed help-seeking leading to SRH issues becoming more serious (e.g., sexually transmitted infections (STIs) being transmitted to others or unmanaged pregnancy) [11, 21].

Recent research reinforces this connection between 1.5 generation migrants’ cross-cultural standpoint, sexuality and SRH. For instance, one paper compared sexual partner risk among Latino adolescents (1.5, 2nd and 3rd generation) in San Francisco [14]. The study found that 1.5 generation migrants were more likely to be involved with risky sexual partner (e.g., one who was involved in gangs and/or drugs) and less likely to report it. Other research [23] which looked at the health of adolescents in the US examined sexual behaviors across migrant generations and found that 1.5 and 2nd generation migrants were less likely than their 1st and 3rd generation counterparts to use birth control. Further, compared to 3rd generation migrants, 1st and 1.5 generation Latinas were less likely to report sexual intercourse. These authors also note the importance and influence of acculturation processes and generation on migrants’ decision-making regarding SRH protective behaviours. A systematic review examining the correlates and predictors of sexual health among adolescent Latinas in the United States [24] discussed cross-generational research. The authors indicated that while some studies attempt to capture the construct of SRH in a multi-dimensional way the concept tends to be reduced to language preference or migrant generation without the exploration of more nuanced understandings. They emphasised that in order to better understand the relationship between acculturation and SRH more nuanced measures are therefore required.

## Methods

This paper is part of a larger project which aimed to investigate the role of culture in constructions of SRH and SRH help-seeking from the perspective of 1.5 generation migrants. Using a mixed methods approach (i.e., questionnaire, Q methodology and interview) the larger study sought to define the key aspects of one’s culture and its messages about sexuality that shape how people within this cohort understand and experience SRH. This paper will focus on the results of the Q methodology portion of the project.

Q methodology allows for the sampling of subjective viewpoints, and can assist in identifying patterns, including areas of difference or overlap, across various perspectives on a given phenomenon [25]. Q methodology can be “described as ‘qualiquantilogical’ combining elements from qualitative and quantitative research traditions [26]. Watts and Stenner [25] indicate five steps for conducting a Q methodology study these include: 1) developing the concourse, 2) developing the Q set, 3) selection of the P set, 4) Q sorting, and 5) Q analysis and interpretation.

### Concourse development and item sampling: Q set

The concourse represents thoughts and opinions or verbalisations about the subject being studied. To develop the concourse a literature review was conducted regarding migrants’ constructions of SRH and help-seeking in Australia. In addition, related materials from online newspapers, websites and clinical guidelines were reviewed to include constructions of SRH not captured in the literature review.

Semi-structured interviews were also conducted with two 1.5 generation migrants (1 female Indo-Australian and 1 male Nigerian-Australian). A total of 120 items were pooled from these steps. Included items were then grouped under four themes: the role of culture on experiences of SRH; constructions and understanding of SRH; the healthcare system and SRH help-seeking behaviour; and external perceptions of migrant sexuality. Within each theme statements were again refined until a final set of items was prepared. This was performed with the purpose of ensuring that all aspects of the topic were covered and not inclined towards a particular viewpoint [27]. The final phase consisted of refining the combined pool of statements with the second, third and fourth authors which involved revision of statements, deletion of duplicates and the addition of other items. Finally, the items were piloted with six volunteers. Following their feedback, the Q set was finalised and included 42 statements that broadly represented migrant constructions of SRH and help-seeking in Australia (see Table 3 in the Results section).

### Selection of participants: P set

With the help of community stakeholders 1.5 generation migrants were recruited via advertisements posted at seven Western Sydney University campuses and surrounding off-campus venues (e.g., major shopping malls, churches, transport stations, restaurants, multicultural and resettlement support centres). This was done in an effort to strategically engage participants from several suburbs within the Greater Western Sydney region to ensure that the Q sorts collected were from as many ethnocultural groups as possible. Following an online survey examining 1.5 generation migrants’ experiences of SRH and help-seeking, 42 out of 112 participants agreed to take part in the Q sort activity. The other 70 declined to participant due to personal time constraints. According to Rogers [28], a sample of 40–60 participants is sufficient to establish the existence of particular construct systems.

### Q sorting

**Fig. 1:**
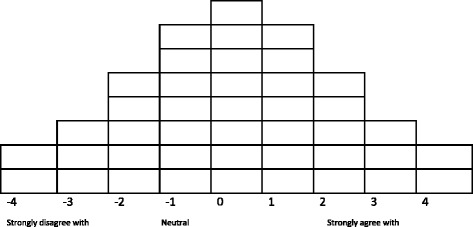
Q Sorting Grid

Participants performed the Q sorting task in three phases using web-based software Q-Assessor [29]. First, participants were instructed to rank-order the 42 statements beginning with those they most agreed with (+4) to those they most disagreed with (−4). To do so participants allocated statements into three clusters: Agree, Disagree, Neutral. Participants then refined each cluster of statements and sorted them in to a quasi-normal distribution sorting grid (Fig. 1) - the standard tool for Q sorting [30]. After the sorting process, participants were asked to answer a series of closed-ended questions to gather socio-demographic data and understand the extent to which their ethnocultural affiliations influenced their identity. This information helped to contextualise the results during factor analysis and interpretation.

### Analysis and factor interpretation

In order to identify perspectives shared by participants, a bi-person factor analysis was performed – a commonly applied statistical method in Q methodology. Factor analysis begins with the calculation of correlation matrix which reveals the degree of agreement or disagreement between the sorts or the similarity or dissimilarity in the views of the Q sorters. From this matrix, initial sets of factors were extracted using the centroid method. The extracted factors were then subjected to varimax rotation; varimax positions factors so that it maximizes the amount of study variance explained [25]. It also provides a more manageable factor solution based on the majority perspectives [25]. Following rotation, factors for interpretation were selected if they fulfilled two criteria: 1) having at least two sorts significantly loading upon a factor and, 2) eigenvalues greater than one [31]. Consequently, a seven-factor solution became the best conceptual fit in this study (see Table 3 Q-set statements and factor array). For those participants, whose responses loaded onto a factor, their responses to the post-Q questions were examined to assist in identifying the meaning of each factor.

## Results

The P set consisted of 1.5 generation migrants who ranged in age from 18 to 39 (23 women, 17 men, and 1 other gender; M_age_ = 22.8, SD = 4.4). Migrants had arrived in Australia as follows, Pre-1999 (4), 2000–2005 (19), 2006–2010 (11) and 2011–2015 (7). The majority migrated from sub-Saharan Africa (18: 42.8%) with the others migrating from South East Asia (8), East Asia (4), Eastern Europe (4), Western Europe (3), the Middle East (2) and the Americas (1). Age upon arrival to Australia ranged from 5 to 19 years with the majority (22: 52.3%) having arrived between the ages of 11 and 15. Other participants arrived between the ages of 0–5 (1), 6–10 (12) and 16–19 (6). Thirty-five (83.3%) participants came to Australia with their immediate family members, 5 came with extended family and 2 came alone. With regards to religion, 23 (54.7%) identified as Catholic/Christian, 7 as Islamic, 2 as Hindu, 2 as Greek Orthodox, 1 as Druze, and 5 indicated that they did not follow any religion. The majority of participants (26: 61.9%) spoke a language other than English at home and 16 only spoke English at home. Table 1 shows the socio-demographic data for participants defining each factor.

**Table 1:** P-set socio-demographic data

Variable		Frequency (%)						
		Factor A *n* = 7	Factor B *n* = 6	Factor C *n* = 4	Factor D *n* = 6	Factor E *n* = 4	Factor F *n* = 8	Factor G *n* = 2
Sex	Male	2 (28%)	3 (50%)	1 (25%)	5 (83%)	1 (25%)	3 (37.5%)	1 (50%)
	Female	4 (57%)	3 (50%)	3 (75%)	1 (16.6%)	3 (75%)	4 (50%)	1 (50%)
	Other	1 (14%)	0	0	0	0	1 (12.5%)	0
Mean Age (SD)		20.4 (2.1)	21.5 (2.2)	24.8 (3.6)	24.2 (7.4)	20.5 (1.7)	25.1 (3.4)	22.5 (3.5)
Relationship Status	Single	7 (100%)	6 (100%)	2 (50%)	5 (83%)	3 (75%)	5 (62.5%)	1 (50%)
	Married	0	0	2 (50%)	1 (16.6%)	0	2 (25%)	1 (50%)
	Other	0	0	0	0	1 (25%)	1 (12.5%)	0
Attracted to	Male	5 (71%)	4 (66.6%)	3 (75%)	0	3 (75%)	3 (37.5%)	1 (50%)
	Female	2 (29%)	2 (33.3%)	1 (25%)	5 (83%)	1 (25%)	3 (37.5%)	1 (50%)
	Both	0	0	0	1 (16.6%)	0	2 (25%)	0
Participants with Children: and number)		0	0	1:2 (25%)	0	0	1:1 (12.5%)	1:1 (50%)
Year entered Australia	Pre-1999	0	1 (16.6%)	1 (25%)	1 (16.6%)	1 (25%)	1 (12.5%)	0
	2000–2005	4 (57%)	1 (16.6%)	1 (25%)	1 (16.6%)	2 (50%)	5 (62.5%)	1 (50%)
	2006–2010	3 (43%)	2 (33.3%)	2 (50%)	3 (50%)	0	1 (12.5%)	0
	2011–2015	0	2 (33.3%)	0	1 (16.6%)	1 (25%)	1 (12.5%)	1 (50%)
Age upon arrival to Australia	0–5	0	0	0	0	1 (25%)	0	0
	6–10	4 (57%)	2 (33.3%)	2 (50%)	0	1 (25%)	2 (25%)	0
	11–15	3 (43%)	2 (33.3%)	2 (50%)	4 (66.6%)	1 (25%)	5 (62.5%)	2 (100%)
	16–19	0	2 (33.3%)	0	2 (33.3%)	1 (25%)	1 (12.5%)	0
Region of origin	Sub-Saharan Africa	4 (57%)	3 (50%)	2 (50%)	0	1 (25%)	0	1 (50%)
	Western Europe	0	0	1 (25%)	2 (33.3%)	0	6 (75%)	0
	Eastern Europe	1 (14%)	1 (16.6%)	0	3 (50%)	0	0	0
	Middle East	0	0	0	0	1 (25%)	1 (12.5%)	0
	South East Asia	1 (14%)	2 (33.3%)	0	0	1 (25%)	1 (12.5%)	0
	East Asia	1 (14%)	0	1 (25%)	0	1 (25%)	0	1 (50%)
	Americas	0	0	0	1 (16.6%)	0	0	0
Religion	Islamic	2 (28%)	1 (16.6%)	0	0	0	5 (62.5%)	0
	Catholic/Christian	4 (57%)	3 (50%)	3 (75%)	2 (33.3%)	2 (50%)	2 (25%)	2 (100%)
	Greek Orthodox	0	1 (16.6%)	0	1 (16.6%)	0	0	0
	Hindu	0	1 (16.6%)	0	0	1 (25%)	0	0
	Non-religious	1 (14%)	0	1 (25%)	2 (33.3%)	0	1 (12.5%)	0
	Other	0	0	0	1 (16.6%)	1 (25%)	0	0
Language spoken at home	Only English	4 (57%)	1 (16.6%)	3 (75%)	2 (33.3%)	1 (25%)	4 (50%)	0
	Additional Language	3 (43%)	5 (83%)	1 (25%)	4 (66.6%)	3 (75%)	4 (50%)	2 (100%)
Who they migrated with	Immediate family	7 (100%)	4 (66.6%)	3 (75%)	5 (83%)	4 (100%)	5 (62.5%)	1 (50%)
	Extended family	0	1 (16.6%)	1 (25%)	1 (16.6%)	0	2 (25%)	1 (50%)
	Alone	0	1 (16.6%)	0	0	0	1 (12.5%)	0

The seven factors explained 52.95% of the total variance. Composite reliability coefficients were examined to evaluate the construct validity for each factor, with all the factors fulfilling the minimum acceptable value of > 0.7 indicating that independent factors were identified. Table 2 presents the characteristics of these factors. Factor scores of each statement across all the seven factors are provided in Table 3. Bracket notations are used to show statement ranking within factor arrays: for example, “(28: +4)” indicates that statement 28 was ranked at +4 (strongly agree).

**Table 2:** Factor Characteristics

Characteristics	Factor						
	A	B	C	D	E	F	G
Number of defining sorts	7	6	4	6	4	8	2
Composite reliability	0.966	0.96	0.941	0.96	0.941	0.97	0.889
Eigenvalue	3.78	3.99	3.47	2.61	3.30	3.94	1.12
Explained variance (%)	9.00	9.51	8.28	6.22	7.88	9.39	2.67

**Table 3:** Q-set statements and factor array

	Q-set statements	Factor						
		A	B	C	D	E	F	G
1	If my family or community found out that I had a sexually transmitted infection they would not be very supportive	2	0	−1	0	1	1	−2
2	I would never let my family or community know that I had sex outside of marriage	1	3	−2	−2	1	4	1
3	If I had a sexual and/or reproductive health issue I would have to find a way to go to a clinic without my family or community knowing	−3	2	0	−1	0	2	−2
4	If my family or community found out that I was involved in an unplanned pregnancy they would not be very supportive	−1	−2	−2	−1	2	−1	2
5	If I had a sexual and/or reproductive health issue other people’s perceptions about it would impact how and when I got professional help	2	1	2	−3	1	0	2
6	Going through adolescence and puberty was sometimes difficult because the things I was taught at school or in the media about sexual and reproductive health were different to what my family or community believe	1	0	−2	0	−3	3	0
7	People who move from one country as children and grow up in Australia are often confused about sexual and reproductive health	0	0	4	0	−1	0	−1
8	I want to pass on to future generations the values about sexual and reproductive health held by my culture of origin	−3	−3	3	0	2	−1	2
9	The way that sexual and reproductive health is dealt with in Australia is very different than the way it is understood in the country where I was born	1	−3	1	0	1	3	1
10	In my origin culture openly discussing sexual and reproductive health is encouraged	−2	−4	2	2	−3	−3	−1
11	In my origin culture women have control over their sexual and reproductive health	−4	0	1	1	1	−3	3
12	In Australia people are encouraged to discuss sexual and reproductive health	−2	−1	3	3	4	3	4
13	People who were born in Australia have an easier time with sexual and reproductive health than migrants like me	1	−2	1	2	4	−1	−1
14	Australians can have intimate relationships with whomever they like and no one would mind	0	2	2	3	−1	0	3
15	Culture plays a large role in how people experience sexual and reproductive health	1	3	−1	4	2	4	0
16	Sexual and reproductive health in my culture of origin is a taboo subject	4	1	1	−3	0	1	−4
17	Australia is very conservative about sexual and reproductive health	−1	1	−3	−2	−2	−1	3
18	In my origin culture sexual and reproductive health is perceived of more in terms of women’s or men’s health	0	1	−1	0	1	1	−1
19	I avoid casual sexual encounters because my family or community would think I was disrespecting my origin culture	0	2	−2	−4	3	2	0
20	Australian men and women think of sexual and reproductive health in the same ways	3	−2	−1	1	−1	−1	−3
21	Australian values lead my understanding of sexual and reproductive health	−3	−1	−4	2	−1	2	−2
22	Sexual and reproductive health refers mostly to prevention of and protection from disease	0	4	−3	1	0	−2	−2
23	Sexual and reproductive health refers mostly to means prevention of unplanned pregnancy	−2	−4	0	−1	−2	−3	0
24	Sexual and reproductive health refers mostly to contraception	2	0	−4	−1	−2	−4	−3
25	Sexual and reproductive health is usually something only promiscuous people have to deal with	−4	1	0	−3	−4	−4	1
26	The way that sexual and reproductive health is understood in Australia is very different than the way it is understood in the country where I was born	1	−1	3	−1	3	2	0
27	There are no words in my culture of origin for sexual and reproductive health	−1	−1	2	−1	−1	0	−4
28	Culture plays a large role in how people understand sexual and reproductive health	3	−1	4	4	2	2	2
29	Health care workers are well equipped to deal with the sexual and reproductive health needs of people from my background	1	0	1	1	0	−2	−1
30	Australians can more easily get help for sexual and reproductive health issues than people from my culture of origin	2	−1	−2	1	3	0	1
31	Migrants need more assistance from health services with sexual and reproductive health than people born in Australia	−2	0	1	3	−1	1	0
32	Health care workers have very little knowledge of the beliefs related to sexual and reproductive health within my culture	0	−3	−3	1	0	0	−2
33	Health services provide the anonymity needed to cater to migrants sexual and reproductive health needs	−1	2	0	1	2	1	0
34	Health services provide clients with a choice between a male or female health care provider	−1	2	0	0	0	0	−3
35	Health services cannot do much else to better cater to the sexual and reproductive health needs of migrants	−2	1	0	0	−1	−2	−1
36	Migrants who identify most as being Australian have more sexual and reproductive health issues than other Australians (excluding Aboriginal and Torres Strait Islander peoples)	−1	3	−1	−2	0	−1	0
37	Migrants who identify most with their culture of origin have more sexual and reproductive health issues than other Australians (excluding Aboriginal and Torres Strait Islander peoples)	0	0	1	−2	−4	−1	1
38	Migrants from certain cultures are carriers of sexually transmitted infections	2	−2	2	−4	−2	−2	−1
39	Migrants who do not take on Australian ways of understanding sexual and reproductive health have failed to assimilate	−1	−1	0	−2	−2	−2	1
40	Australians should take on more values about sexual and reproductive health from migrant cultures	0	4	0	2	−3	1	1
41	Migrant sexual and reproductive health needs are quite different from those of non-migrants	3	−2	−1	−1	0	0	2
42	Australians may think that some migrant groups have out-dated ideas about sexual and reproductive health	4	1	−1	2	1	1	4

### Factor A: Struggle between progressive and traditional sexual and reproductive health values

Factor A accounted for 9% of the total variance with the Q sorts of 7 participants defining this factor (see Table 2). Factor A is characterised by comparisons of SRH values in Australia (relatively progressive) and migrants’ culture of origin (relatively traditional). Evidence of this may be that women in their culture of origin do not have control over their SRH (11: −4) while no such gender difference is perceived in Australia (20: +3). Further, participants strongly agreed that Australians view migrants’ ideas of SRH as out-dated (42: +4).. This perspective of traditional versus progressive constructions of SRH is corroborated by the participants’ agreement that within their culture of origin SRH is a taboo subject (16: +4). Participants perspectives on the differences between cultures is also evident given their agreement with the idea that migrant SRH needs are different from those of non-migrants (41: +3) and that health services can do more than they are already doing to cater specifically to migrants’ SRH needs (35:−2). It may be these differences which led migrants to believe that health care workers are minimally equipped to deal with their SRH needs (29: +1). Even so, migrants indicated that if they needed to go to a clinic for an SRH issue they did not feel it necessary to hide this from their community or family (3:−3). Family and community openness was, however, moderate as participants felt that if they had an STI their community and family would not be supportive (1: +2). As such, these participants believed that the way SRH is dealt with in Australia is different than the way it is dealt with in their country of origin (9: +1). Given the relatively more relaxed views of SRH in Australia participants noted that they did not want to pass on to future generations the values about SRH held in their culture of origin (8:−3).

Based on the post-Q questionnaire factor representatives identified strongly with both their culture of origin and Australian culture (71.4%). With the majority being from non-Western countries and identifying as religious these migrants may experience challenges in their efforts to compare, assess, review and re/construct their SRH values.

### Factor B: Experiencing SRH through two cultural lenses

Factor B accounted for 9.51% of the total variance with the Q sorts of 6 participants defining this factor. Factor B demonstrated the influence of culture on SRH constructs within cross-cultural contexts. Socially participants felt that Australians should take on SRH values from migrant cultures (40: +4). Participants also believed that migrants who identify most as being Australian have more SRH problems than other Australians (36: +3). This may be a criticism of Australian culture and its influence on SRH. Criticism is also seen in migrants’ evaluation of their own cultures. Cultural support can be seen in participants’ belief that if confronted with an unplanned pregnancy their family and community would be supportive (4:−2). Even so, there was a moderate sense that going to a clinic for an SRH issue without family or community knowing was preferred (3: +2). It seems that the migrants had a more liberal set of SRH values. This is reflected in the respondents’ belief that how SRH is dealt with in Australia was not very different to their culture of origin (9:−3). They also believed that people born in Australia did not necessarily have an easier time with SRH compared to migrants like themselves (13:−2) or that non-migrants understood SRH differently than they did (26:−1).

The post-Q questionnaire indicates that all exemplars strongly identified with their culture of origin (sub-Saharan Africa (50%), South East Asia (33.3%) and Eastern Europe (16.6%)). This may explain their critique of how SRH is constructed in Australian culture. This is further corroborated as 83.3% strongly agreed or agreed that they had a robust relationship with their community based on their culture of origin.

### Factor C: Importance of the culture of origin

Factor C accounted for 8.28% of the total variance with the Q sorts of 4 participants defining this factor. Factor C exemplars strongly believed that culture played a large role in how people understand SRH (28: +4). These participants were keen to pass on values about SRH from their culture of origin to future generations (8: +3) and indicated that Australian values did not lead their understanding of SRH (21:−4). They also felt that the way in which SRH is understood in their culture of origin and in Australia were quite different (26: +3) with Australia being more liberal in this regard (17:−3). A key difference was having no words in their mother tongue for SRH (27: +2) which would have a significant impact on how SRH was constructed. Further, the potential differences between one’s culture of origin and those within Australia can cause confusion about SRH (7: +4). For example, exemplars moderately agreed that “migrants from certain cultures are carriers of sexually transmitted infections” (38: +2) and that migrants who identify most with their culture of origin have more SRH issues than other Australians (37: +1).

Although exemplars perceived differences regarding SRH between their culture of origin and in Australia there are some aspects of cultural community support which resemble those in Australia. For instance, in the participants’ culture of origin discussing SRH is encouraged (10: +2) and they would not feel uncomfortable if their family or community knew that they had sex outside of marriage (2:−2). If they were confronted with an unplanned pregnancy their community and family would also be supportive (4:−2). Although support is present, the exemplars indicated that their ability to seek professional help for an SRH issue would be impacted by others’ perceptions (5: +2). This hindrance does not seem linked specifically to one cultural context as participants indicated that Australians do not get help for SRH issues more easily than their migrant counterparts (30:−2). Further, help-seeking was not influenced by health care workers’ understanding of SRH within various cultures (32:−3). As such, participants felt that health care workers were moderately well-equipped to handle the SRH needs of people from their own background (29: +1).

The questions posed after the Q sort indicate that half of the exemplars identified most with their culture of origin while the other half were neutral. Although these exemplars see culture as an important factor in constructions of SRH the salience of their culture of origin seems less prominent in their SRH behaviour and help-seeking. With the majority coming from sub-Saharan Africa and being Christian/Catholic their culture of origin was important but may not have been the most important element in their constructions of SRH.

### Factor D: Acknowledging sexual and reproductive freedom

Factor D accounted for 6.22% of the total variance with the Q sorts of 6 participants defining this factor. Factor D describes the role of culture in migrants’ intimate relationships, beliefs about SRH and engagement of health care services. For these participants, culture played a large role both in how SRH is understood (15: +4) and experienced (28: +4). Australian values also led their understanding of SRH (21: +2). Participants felt that Australians can have intimate relationships with whomever they liked and that no one would mind (14: +3). Based on this belief the exemplars did not avoid casual sexual encounters in fear that their family or community would not approve (19:−4) nor did they feel that their family or community would frown upon sex outside of marriage (2:−2). Participants also reported that their SRH help-seeking would not be impacted by the perception of others (5:−3). This way of understanding SRH, and thus, engaging in intimate relationships in Australia was not that different to understandings of SRH in the migrants’ culture of origin (26:−1). This liberal way of interacting with SRH is perhaps a result of being encouraged to discuss SRH within their culture of origin (10: +2) e.

Cultural differences were, however, implied as participants agreed that migrants need more assistance from SRH health services than people born in Australia (31: +3). Exemplars may have felt this way as they indicated that health care workers had limited knowledge about the SRH beliefs within their culture of origin (32: +1) and may not be equipped (29: +1) to handle their needs. Even so these exemplars did not perceive major differences between migrant groups and other Australians. For instance, they disagreed that migrants who identified most with Australian culture had more SRH issues (36:−2), that migrants who do not take on Australian ways of understanding SRH had failed to assimilate (39:−2) and they disagreed that migrants from certain cultural groups were carriers of STIs (38:−4).

The post-Q sort questions indicate that the majority of exemplars identified with the culture and values of their country of origin (83.3% strongly agreed or agreed) and had strong relationships with their family and community as a result of these values (66.6% strongly agreed or agreed).

### Factor E: Cultural differences in sexual and reproductive behaviour

Factor E accounted for 7.88% of the total variance with the Q sorts of 4 participants defining this factor. Factor E describes the impact of culture on SRH related behaviour. For these exemplars, there was quite a difference in the way SRH is understood in Australia and in their culture of origin (26: +3). For instance, they perceived that in Australia people are encouraged to discuss SRH (12: +4) and that people born in Australia have an easier time with SRH than migrants like themselves (13: +4). Part of having an easier time with SRH included the perception that Australians are better able to access support for SRH issues over their migrant counterparts (30: +3). These participants also avoided casual sexual encounters because their family or community would perceive such behaviour as disrespectful (19: +3). Participants’ behaviour moderation was not necessarily in an effort to avoid STIs or unplanned pregnancy (25:−4) but was instead in an effort to avoid disapproval. For example, participants also agreed that their family or community would not be supportive if they had to manage an unplanned pregnancy (4: +4).

While migrants’ SRH behaviours may have been dictated by cultural pressures this did not seem to result in a culture clash between their culture of origin and Australian culture. For instance, participants disagreed that going through puberty and adolescence was difficult as a result of being exposed to differing messages about SRH (6:−3). Exemplars also disagreed that moving from one country to another during childhood/adolescence caused confusion about SRH (7:−1).

Interestingly, the post-Q sort questions show that the majority of the exemplars were neutral or disagreed (75%) with identifying most with the values of their culture of origin and were neutral or disagreed (75%) with identifying most with Australian culture. This contextual information suggests that these migrants did not explicitly retain or denounce the SRH values of their culture of origin nor do they take on the values of their adoptive culture.

### Factor F: Mixed sexual and reproductive health messages

Factor F accounted for 9.39% of the total variance with the Q sorts of 8 participants defining this factor. Factor F delves into the messages which the participants may have been given as they grew up. There was strong agreement that culture plays a large role in how people experience SRH (15: +4) and the ways in which SRH is dealt with in Australia and their culture of origin is very different (9: +3). As such, participants may have decided to take on Australian values which now lead their understanding of SRH (21: +2). The process of making such a decision was however complicated. For instance, participants agreed that going through puberty and adolescence was sometimes difficult because of conflicting SRH messages from school or media and their family or community (6: +3).

However, the content of the messages received from their culture of origin may not have been specific to SRH. For instance, SRH did not simply refer to contraception (24:−4) or linking SRH risks to promiscuity (25:−4). The content may have been more morally bound or religious in nature as exemplars would never let their family or community know that they had had sex outside of marriage (2: +4). Given the variety of messages and ways of understanding SRH, the participants indicated that health care workers may have a hard time managing the needs of migrant populations. Notably, participants perceived that health care workers were not well equipped (29:−2) and health care services did not do enough to cater to the needs of migrants (35:−2). Given the varying messages regarding SRH it is not surprising that health care workers would find it hard to keep up.

The post-Q questionnaire showed that most identified (75%) with the values of their culture of origin with 62.5% being neutral or disagreeing about identifying most with Australian culture. While 75% of the exemplars came from Western Europe the majority identified as religious (62.5% Muslim and 25% Christian/Catholic). As such the impact of multiple messages may have had a confusing effect on their constructions of acceptable SRH behaviour.

### Factor G: Understanding sexual and reproductive health across cultures

Factor G accounted for 2.67% of the total variance with the Q sorts of 2 participants defining this factor. Factor G revealed many similarities between Australian and migrant cultures. For instance, exemplars agreed that in Australia people are encouraged to discuss SRH (12: +4) and that SRH was not a taboo subject in their culture of origin (16:−4). Further, the migrants note that there are many words in their culture of origin to facilitate such discussions (27:−4) and that that women have control over their SRH (11: +3). Participants agreed that Australians may believe that some migrant groups have out-dated ideas about SRH (42: +4) but that Australia was in fact quite conservative with regards to SRH (17: +3). As such, these exemplars felt that young migrants are not confused about SRH (7:−1) which may be linked to their belief that failure to adopt Australian values regarding SRH is an indication of a migrant’s failure to assimilate (39: 1). This is further corroborated by participants’ agreement that migrants who identify most with their culture of origin experience more SRH issues than other Australians (37: +1).

Participants believed that others’ perceptions would have an impact on how and when they got professional help for an SRH issue (5: +2) – perhaps because they believed that SRH is something promiscuous people generally have to contend with (25: +1). Although their family or community would be very supportive if they found out the participant had contracted an STI (1:−2) this was not so if the participants were involved in an unplanned pregnancy (4: +2). Considering their perception that both their culture of origin and Australian culture are equally conservative on these matters, limitations in community or family support were no different in either context.

The post-Q sort questions indicate that all exemplars identified strongly with their culture of origin as well as Australian culture. As they deemed their culture of origin and Australian culture to be similar, there would be little SRH related culture clash.

## Discussion

This study investigated the role of culture in constructions of SRH and help-seeking from the perspective of 1.5 generation migrants. The results show a range of processes involved in comparing, assessing, reviewing and re/constructing SRH and help-seeking between and across cultures. For some migrants the meaning of SRH constructs changed when in a different cultural context [32]. On the one hand, some participants experienced significant difficulty integrating new cultural values. As noted by Dune et al. [11] challenges may surface as migrants try to make meaning of and establish value sets which are congruent with their identity, beliefs and experiences (see also [10]). On the other hand, others found it relatively easy to make a choice between which SRH constructs to retain, absorb, integrate or reconstruct (see also [33]). The results reinforce that the there is no single construction of SRH and help-seeking even among young migrants. Further, not all migrants’ constructions are that different from those held in Australia. This challenges the populous belief that Australia is a very liberal and secular [34] country while other (especially non-Western cultures) are colloquially described as more conservative. As such, Australian society, policy and health care services may overlook their own conservatism around SRH and therefore miss opportunities to appropriately engage with migrant SRH care [35]. The results from the post Q questionnaire demonstrate the importance of participants’ sociodemographic characteristics as an indication of the acculturative context in which these results are interpreted. These characteristics also provide direction for future research, policy and practice.

With the majority of participants being religious (83.3%) it may not be their ethnic culture which drove their understandings of SRH but instead their religious values. Through this lens extracting or cherry-picking which SRH constructs could stay and which could go may not have made sense as they were intertwined with deeply held religious beliefs. Within the context of Islam for example Smerecnik, Schaalma, Gerjo, Meijer and Poelman [36] explain that sexuality is not restricted to procreation as in most other monotheistic religions. Instead, sexuality is considered to be an expression of spirituality. Therefore, for those migrants who have come from more religious societies Australia’s relatively secular and liberal society may cause confusion when migrants try to decide how to engage with SRH values in the context of their religion (e.g., Catholic/Christian) versus their cultural identity (e.g., Australian + culture of origin). This may especially be the case when young migrants do not see themselves as culturally different, but who may be quite different in their adherence to religious culture. Even amidst the possible confusion of mixed messages religion may serve as a grounding point for young migrants in their efforts to understand and establish their sexuality as it relates to their cultural and social identities. This is of particular relevance to the Greater Western Sydney area in Australia which has many pockets of cultural concentration which allows migrants to stay connected to their culture such as their ethnicity, community, language and religion [3]. If these migrants integrate into a similarly religious environment in Australia the messages they received within their country of origin and Australia may not be significantly different or result in any ‘cultural’ clash per se. It is therefore important that religiosity be investigated for its influence on constructions of SRH and their impact on young migrants’ help-seeking behaviours. It should also be noted that religious organisations readily engage in discussions about SRH with their young constituents. A better understanding of the educational approaches of these organisations and the role they play in the development of SRH messages during adolescence and puberty for young migrants may illuminate avenues to educating about SRH through culturally-appropriate channels.

The age of arrival in Australia may have a significant influence on the ways in which participants re/constructed SRH and help-seeking. For instance, participants who arrived before the age of 10 may have little to no understanding of SRH constructs of their country of origin [37]. Those who arrived between the ages of 11 and 15 (the majority of participants) may have developed some sense of SRH in the context of their culture of origin therefore having a point of reference for Australian SRH constructs. Research indicates that migrants in this age group may have difficulty consolidating the SRH messages they receive at home and those that are presented in Australian society [11]. The consequence of such discrepancy may manifest in difficulties managing both intimate and familial relationships [11]. While the constructs held by this group may have more permeable parameters the constructs of those who arrived between 16 and 21 years of age may be more rigid and distinct [38]. Given their older age upon arrival these migrants have more mature cognitive abilities and are better able to see the parameters around their culture of origin and Australian culture and therefore able to decide whether to integrate the two [39]. A closer look into age upon arrival can provide more information in the development of policies and sex education programmes for young migrants.

This study unearthed several interesting and previously undocumented findings with regards to constructions and understandings of SRH across cultures. The study has also highlighted key areas which require further consideration and investigation. For instance, SRH values include a very broad range of concepts which could mean and comprise of different things for different migrant groups. Further research on the content of these values would be beneficial and would provide a better sense of which values from migrants’ country of origin are difficult to integrate with Australian values and the impact this may have on SRH help-seeking and outcomes.

The purposeful nature of the sampling strategy helped to achieve a varied sample with the aim of capturing perspectives from various ethnic, religious and migration histories. However, the country of origin of the sample was not proportional as most participants were from sub-Saharan Africa. In addition, the majority of participants were Catholic or Christian which may not reflect many 1.5 generation migrants who do not prescribe to Christianity. This cultural similarity may mean the full breadth of cross-cultural constructions of SRH have yet to be explored. Thus, generalisations cannot be made about the different perspectives among such groups, and further study is recommended to assess the effect of country of origin and religious background on constructions of SRH amongst young migrants in Australia.

Although participants of this study were recruited from a number of Western Sydney suburbs this was done in relation to seven Western Sydney University campuses and surrounding off-campus venues. As a result, the participants are likely to have been university students or staff and therefore engaged with higher education. As such, the sample may not be representative of the many 1.5 generation migrants who may not have high levels of education. With lower levels of education come lower levels of health literacy [40]. Consequently, participants’ perspectives on health care services and the engagement of those services with migrants may be influenced by their increased ability to scrutinise, navigate and mediate their experiences within the Australian health care system compared to other groups of migrants. Further 70 participants of the initial survey chose not to participate in the Q sorting activity which may have further biased the sample. Expansion of this study to include a broader variety of 1.5 generation migrants is therefore recommended.

## Conclusion

A range of seven distinct perspectives that indicate variation in 1.5 generation migrants’ constructions of SRH and help-seeking were revealed using Q methodology. The data highlights that young migrants vary in their acculturation journeys. Such variation may have less to do with culture or even age upon arrival and more to do with religiosity. This is important to consider as migrants are expected to culturally adapt, in this case, to “Australia’s way of life” [41] while one’s connections with their religious beliefs and practices are not expected to change. In that sense, it may be easier to adapt one’s culture as many aspects of ‘home’ simply do not exist in the adoptive country (e.g., political, economic, judicial, health and social systems). However, one’s religion (and most of its contents) is portable so can be practiced as it was in one’s country of origin. Perhaps it is here that a culture clash exists.

The data highlights potential complications to the development and delivery of cross-cultural SRH education and services. As such, further research with migrant youth can provide insight about effective strategies for cross-cultural, intercultural and/or multicultural SRH education. This would be used to inform the development of a responsive, flexible and adaptable model to address this gap in migrant resettlement service delivery. It would also assist migrant parents and youth in exploring, discussing, reframing and reconstructing SRH in an Australian context. Australia’s unique experience of multiculturalism, relatively short history of migration as well as its legal and political frameworks requires that Australian migrant communities and organisations be central in the identification and exploration of keys issues and strategies relevant to the Australian context [42].

## Abbreviations

SRH: Sexual and reproductive health
STIs: Sexually transmitted infections
